# Supplementary material for: Quantitative Determination of Ethylene Using a Smartphone-Based Optical Fiber Sensor (SOFS) Coupled with Pyrene-Tagged Grubbs Catalyst
Source: Biosensors (Basel). 2022 May 10;12(5):316. doi: 10.3390/bios12050316 (PMC9138739; doi:10.3390/bios12050316)
Supplement: Supplementary file 1 [file biosensors-12-00316-s001.zip › biosensors-1696069-supplementary.pdf]

Supporting Information

Quantitative Determination of Ethylene Using a Smartphone-Based Optical Fiber Sensor (SOFS) Coupled with Pyrene-Tagged Grubbs Catalyst

Xin Yang [1,†], Justin Lee Kee Leong [1,†], Mingtai Sun [1,2,*], Linzhi Jing [1,3], Yuannian Zhang [1], Tian Wang [4], Suhua Wang [2] and Dejian Huang [1,3,*]

[1] Department of Food Science and Technology, National University of Singapore, 2 Science Drive 2,
Singapore 117542, Singapore; xinyang@u.nus.edu (X.Y.); justinleekl@gmail.com (J.L.K.L.); e0047517@u.nus.edu (L.J.); zhangyuannian@u.nus.edu (Y.Z.)

[2] School of Environmental Science and Engineering, Guangdong University of Petrochemical Technology, Maoming 525000, China; wangsuhua@ncepu.edu.cn

[3] National University of Singapore (Suzhou) Research Institute, 377 Linquan Street, Suzhou 215123,
China

[4] Department of Chemistry, National University of Singapore, 3 Science Drive 3,
Singapore 117543, Singapore; tian.wang91@u.nus.edu

* Correspondence: mtsun@gdupt.edu.cn (M.S.); dejian@nus.edu.sg (D.H.)

† These two authors contributed equally to this work.

* To whom correspondence should be addressed:
mtsun@gdupt.edu.cn (M. Sun); dejian@nus.edu.sg (D. Huang)

Table of contents

I. ADDITIONAL EXPERIMENTAL PROCEDURES 2

II. TABLE & FIGURE..3

I. ADDITIONAL EXPERIMENTAL PROCEDURES

Procedures for preparing interfering gases.

Sodium sulfite (Na_2SO_3) was freshly prepared with a stock solution concentration of 10.0 mM in PB 7.4 solution. SO_2 gas was prepared from the reaction of Na_2SO_3 with concentrated H_2SO_4 in a 25 mL round bottom flask. Carbon dioxide (CO_2) was prepared by diluting the pure CO_2 gas to the target concentration in a 25 mL round bottom flask. NO_2 gas was prepared from the reaction of pure NO with air in a 25 mL round bottom flask. H_2S gas was prepared from the addition of Na_2S with PB 7.4 solution in a 25 mL round bottom flask. NH_3 gas was prepared from the reaction of NH_4Cl with $Ca(OH)_2$ in a 25 mL round bottom flask. Generally, 2 mL of these gas samples were slowly bubbled 1 mL dichloromethane before addition of 30 μL PYG probe stock solution and shaken for 3 min. The RGB values were recorded using a CMOS sensor with ISO 800, exposure rate 1/30s and 1440p.

II. TABLE and FIGURE

Table S1. RGB values of the PYG solution bubbled with different concentrations of ethylene.

Concentration of Ethylene (ppm)	R_μ	G_μ	B_μ
500	1.33	36.00	107.67
400	1.00	49.67	116.33
350	1.33	72.00	143.00
300	1.67	73.67	139.33
250	1.33	79.00	134.67
200	1.33	84.00	138.00
150	1.33	82.00	120.33
100	1.67	82.67	115.00
75	1.67	85.33	114.33
50	1.67	86.67	111.33
37.5	1.33	90.00	111.67
25	1.00	98.00	117.33
20	1.33	85.00	100.67
15	2.00	74.00	87.67
0	1.00	65.00	74.00

Table S2. $(B/G)/(B/G)_0$ values of the PYG solution bubbled with different interfering gases and ethylene at 100 ppm.

Species	$(B/G)/(B/G)_0$
Blank	1.233
Ethylene	1.029
MeOH	1.022
EtOH	1.000
1-prop	0.992
H2O	1.009
EA	1.009
Tol	1.001
THF	1.222

Table S3. $(B/G)/(B/G)_0$ values of the PYG solution bubbled with different interfering gases and ethylene at 100 ppm.

Gas Species	w/o Ethylene	w Ethylene
Blank	1	1.225
SO_2	1.029	1.235
CO_2	1.020	1.227
NH_3	0.989	1.184
NO_2	1.008	1.224
H_2S	1.012	1.222

Table S4. Ethylene concentration of avocados in an airtight jar incubated over 8 hours.

Time	B/G_1	B/G_2	B/G_3	Mean	STDEV	$(B/G)/(B/G)_0$	[Ethylene] (ppm)
0	1.132	1.145	1.135	1.137	0.009	1.000	0
2	1.179	1.187	1.184	1.183	0.004	1.041	19.112
4	1.246	1.235	1.232	1.238	0.008	1.089	41.564
6	1.280	1.284	1.296	1.287	0.008	1.132	61.730
8	1.318	1.326	1.330	1.325	0.006	1.165	77.337

Table S5. Ethylene concentration of apples in an airtight jar incubated over 8 hours.

Time	B/G_1	B/G_2	B/G_3	Mean	STDEV	$(B/G)/(B/G)_0$	[Ethylene] (ppm)
0	1.321	1.327	1.327	1.325	0.005	1.000	0
2	1.379	1.350	1.357	1.362	0.015	1.027	12.464
4	1.383	1.407	1.393	1.395	0.012	1.050	23.529
6	1.422	1.415	1.418	1.418	0.003	1.069	32.353
8	1.435	1.433	1.448	1.439	0.008	1.084	39.654

Table S6. Ethylene concentration of bananas in an airtight jar incubated over 8 hours.

Time	B/G_1	B/G_2	B/G_3	Mean	STDEV	$(B/G)/(B/G)_0$	[Ethylene] (ppm)
0	1.241	1.236	1.232	1.236	0.004	1.000	0
2	1.284	1.297	1.299	1.293	0.008	1.046	21.475
4	1.333	1.328	1.333	1.332	0.003	1.077	36.161
6	1.381	1.379	1.375	1.378	0.003	1.115	53.840
8	1.435	1.443	1.429	1.436	0.007	1.161	75.596
